# Mapping Turnaround Times (TAT) to a Generic Timeline: A Systematic Review of TAT Definitions in Clinical Domains

**DOI:** 10.1186/1472-6947-11-34

**Published:** 2011-05-24

**Authors:** Bernhard Breil, Fleur Fritz, Volker Thiemann, Martin Dugas

**Affiliations:** 1 Institute of Medical Informatics, University of Münster, Domagkstraße 9, 48149 Münster, Germany

**Keywords:** Turnaround time, hospital information systems, cycle time, process time, timeline

## Abstract

**Background:**

Assessing turnaround times can help to analyse workflows in hospital information systems. This paper presents a systematic review of literature concerning different turnaround time definitions. Our objectives were to collect relevant literature with respect to this kind of process times in hospitals and their respective domains. We then analysed the existing definitions and summarised them in an appropriate format.

**Methods:**

Our search strategy was based on Pubmed queries and manual reviews of the bibliographies of retrieved articles. Studies were included if precise definitions of turnaround times were available. A generic timeline was designed through a consensus process to provide an overview of these definitions.

**Results:**

More than 1000 articles were analysed and resulted in 122 papers. Of those, 162 turnaround time definitions in different clinical domains were identified. Starting and end points vary between these domains. To illustrate those turnaround time definitions, a generic timeline was constructed using preferred terms derived from the identified definitions. The consensus process resulted in the following 15 terms: admission, order, biopsy/examination, receipt of specimen in laboratory, procedure completion, interpretation, dictation, transcription, verification, report available, delivery, physician views report, treatment, discharge and discharge letter sent. Based on this analysis, several standard terms for turnaround time definitions are proposed.

**Conclusion:**

Using turnaround times to benchmark clinical workflows is still difficult, because even within the same clinical domain many different definitions exist. Mapping of turnaround time definitions to a generic timeline is feasible.

## Background

Health care processes are difficult to define because of their complexity [1]. Assessing time definitions in clinical processes can help to analyse workflows in hospital information systems (HIS) and to identify weak points [2]. Due to increasing costs in health care it is important to improve the efficiency of clinical workflows.

When analysing process times, it is important to be aware of the different definitions used for time intervals. One of the most common measures of laboratory or pathological services is the turnaround time (TAT) which has frequently been used since 1980 to quantify the time for laboratory tests in an objective manner [3]. The first reference dates from 1971 and describes TAT as the time interval between electrocardiogram printing and placement of the printout in the patient chart [4]. In the laboratory workflow TAT is an important indicator of performance [5] and is even seen as a "necessary condition for (...) trust between patient and physician" [6].

Publications about report TAT in radiology workflow [7], TAT for processing medication orders [8] or patient cycle time [9] demonstrate that these process indicators are not limited to pathology or laboratory services. These parameters can be used for HIS monitoring and benchmarking especially with respect to process descriptions and assessments. However, in the available literature many different definitions for TAT are used. Starting and end points for specific processes depend on several factors such as hospital departments (e.g. laboratory, pathology, emergency department), analysed subjects (e.g. patients, specimens), included activities and priority, which all result in varying points in time and also different units of measurement from seconds over hours to days.

On the one hand TAT, for example, can be defined as "time from receipt of the specimen" until "time of availability of the result" (laboratory TAT) [10] as well as "time from the physician's request" until the "time the physician views the result" (total TAT) [11]. On the other hand there are the drug turnaround time [12] and medication TAT [13], which describe the same time interval. Being a common problem not only in medicine, those parameters are used as synonyms and homonyms. Using different TATs is reasonable to measure and evaluate certain aspects and systems, but only if they are precisely defined. Fuzzy definitions and the use of synonyms and homonyms make it difficult to compare processes between hospitals. In this context we conducted a literature review to detect similarities as well as varieties in order to facilitate benchmarking.

Most of the previous literature reviews regarding TAT are focused on laboratory or pathological departments. Manor had analysed literature concerning TAT in clinical laboratories from 1989 until 1999 to evaluate methods in order to improve the processes in clinical laboratories [14]. At that time she compared pneumatic tubing systems with decentralized testing, satellite laboratory, point-of-care testing and computer technology. Currently, most processes are supported by computer technology. This is the reason why Georgiou and Westbrook analysed only computerised physician order entry systems (CPOE) in their literature reviews of 2006 [15,16]. They assessed the general impact of CPOE and focused on the three phases of pathological services "preanalytical", "analytical" and "postanalytical", while comparing designs and results of recent articles. However, this review is also limited to pathological services and does not include other process time definitions like drug TAT or time for diagnosis. Another literature review, concerning computerization, analysed general time efficiency as a result of the use of electronic health record [17]. In his review of laboratory TAT in 2007 Hawkings reveals the different steps of a testing cycle from ordering to interpretation and action. He states that data for extra-laboratory activities are needed, because often non-analytical delays are prolonging the total TAT [5].

In a recent review [18] Schimke compared central laboratory testing to point of care testing and argues that there is only limited potential for further shortening of the intra-laboratory TAT. Increased patient benefit can only be achieved by earlier clinical decisions, based on the results which require better integration of pre- and post-analytical phases (ultimately point of care testing). In one of the latest publications in this field TAT was compared while assessing the impact of CPOE systems on clinical workflow [19].

There is a vast range of literature concerning TAT for pathological and laboratorial services, which focus on design of CPOE systems, results, usability and quality effects. There are also papers in which the domains are overlapping, for example, when laboratory point of care testing was performed in the emergency department to improve their workflow [20,21]. However, the literature concerning not only process analyses but containing also precise definitions of process indicators in various domains is limited. The purpose of this paper is to review TAT definitions, which are used to analyse clinical workflows in different medical domains in order to measure effects of the use of information technology.

Our objectives in this review are to:

1) collect relevant literature concerning process times in clinical workflows,

2) analyse the existing TAT definitions and the related domains and

3) summarise these TAT definitions and map them to a generic timeline.

## Methods

### Constraints

The term information system in our analysis is constrained to clinical information systems with a direct impact to an electronic health record. Therefore we did not include systems which are primarily used for billing, enterprise resource planning or data warehousing. Consequently, the considered groups of users are hospital staff and patients. We did not distinguish processes for inpatients and outpatients, as generic TAT definitions are very similar, for example regarding laboratory processes.

### Literature collection

A literature review was carried out to summarise process time indicators in studies having been published until 31.12.2009. The main search was based on the Medline database via Pubmed [22]. In addition, the reference lists from relevant articles and additional articles by key authors were also reviewed. Three reviewers with previous scientific experience in HIS and clinical processes selected and reviewed all papers (BB, FF, VT). The decision about including or excluding a paper in our analysis was based on consensus discussion.

We experimented with several query approaches (for example MeSH-terms like "process assessment (health care)/methods" or free text "process time"). As these queries resulted in a great amount of hits, which proved to be very unspecific, we identified the term "turnaround time" to be the most specific search criterion.

Our approach was to search for the term "turnaround time" in title and abstract. Then we limited the results to those having an abstract. We removed all papers not written in English or German. As our main interests were the effects of the use of information technology, we further removed those not being related to the use of computerised systems and those with a strong biological and/or medical focus (for example new laboratory test methods).

**Figure 1:**
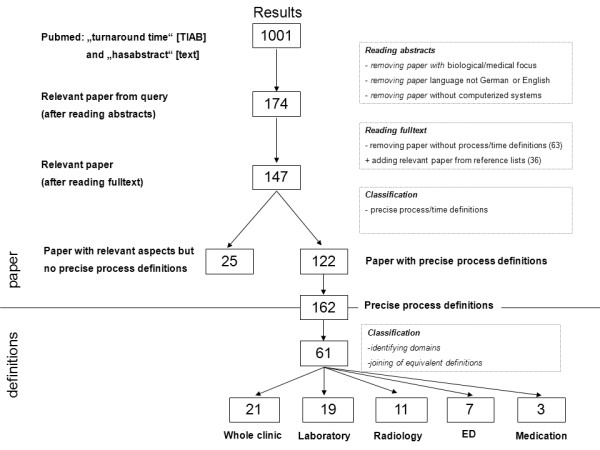
**Literature Review**. Starting with 1001 results coming from a Pubmed research we identified 174 relevant papers. In our review we then used 135 papers of which 122 contained a total number of 162 precise definitions. After classifying them according to their starting and end points we ended up with 61 definitions covering five clinical domains (ED = Emergency Department).

During the full text review we removed all papers that did not include process time definitions and we added relevant papers found through a review of referenced publications. This process is also illustrated in Figure 1.

### Analysis process

The resulting relevant articles were reviewed according to process indicators, related to time in different medical domains. We grouped them into those having a time definition with starting and end points and those having relevant aspects but no precise definitions. From the first group we extracted information about the short and long text of the definitions, the starting and end points of the process indicators, the units of the measured time, the domains and the year of publication. This information was aggregated in a separate list of definitions, keeping the original articles as references. Articles from the second group were used for background information.

### Summarizing definitions

We collected information about TAT definitions concerning their abbreviations, starting and end points as well as measured time units (days, hours and minutes). In order to depict these TATs a suitable timeline is needed. As TAT intervals usually span over different points in time, we selected the most commonly used and best defined terms for corresponding points in time through a consensus process. For example, in a study by Ramaswamy the report turnaround time (RTAT) includes four steps [23]. Based on our harmonisation, a timeline was designed to bring all identified process times in a sequential order and to show which steps are included.

## Results

### Collection and analysis process

As illustrated in Figure 1 the PubMed search resulted in 1001 publications about TAT. After reading the abstracts and removing those not relevant according to our inclusion criteria, 174 publications were left, 63 of which were removed after having read the full text and another 24 were added as a result of reviewing reference lists of key articles. The remaining 135 articles were agreed upon by all three reviewers to be relevant with respect to our aim. Thirteen of them did not contain precise enough definitions of the starting and end points of the process and were therefore not classified in the list of definitions. Out of the other 122 articles all relevant information according to our analysis strategy was extracted and resulted in 61 definitions of TAT [24-145]. Many of these measures were not precisely specified and had to be deduced from the context. To classify the resulting definitions, we chose the following five different clinical domains, which we considered being appropriate for our further analysis: laboratory, radiology, medication, emergency department and whole clinic. The domain "whole clinic" contains processes of different departments such as operation theatre. Most definitions were therefore found in the domain whole clinic (21) followed by definitions concerning laboratory (19), radiology (11), emergency department (7) and medication (3). The full table with all definitions and their references can be found in the supplement (see Additional file 1).

### Summarizing TAT definitions

While analysing and collecting TATs we identified terms which range from admission to the final report after discharge. Some expressions were used synonymously for starting and end points (e.g. "biopsy" and "sampling"). In total, we identified 15 points in time regarding the description of the process independent from the respective user, although some are predominantly relevant for TATs in a specific domain (e.g. "receipt of specimens in laboratory" in a laboratory).

Although there are some processes which start before the patient is admitted to a hospital, we chose admission as the first starting point, as it is rather difficult to measure pre and post clinical times. We considered the admission as a synonym for the term hospitalization as well as for the arrival at a department. The next step after admission is typically the anamnesis in which the physician orders or requests a service. This service may be the examination of the patient or the starting point of anaesthesia and an operation procedure. In a laboratory context this examination can be a biopsy (also called the collection of specimen/sampling) and in radiology the examination can be the radiography. After the examination, which ends with the patient or physician leaving the operation/examination room, the specimen reaches the laboratory. This point in time is relevant in a laboratory context and very often measured and documented in the laboratory information system (LIS). The next commonly described point in time is the completion of the procedure or examination. In a laboratory context this is the time, when a result is available. This can be considered similar to the completion of an X-ray or any imaging procedure, which is completed and has to be interpreted in the next step. This interpretation ends when a first diagnosis or result is available. Dictation and transcription (which is a synonym for typing) are commonly used in radiology, whereas verification or reviewing of the preliminary results is also done in laboratories and other domains. The next important step is the availability of the report, in many cases containing the finalized and verified diagnosis, which is then delivered back to the requester. This point in time ranges from the delivery of a report to the arrival of blood components or medications. In some papers the time when viewing the delivered report was also considered as a relevant step. Then following is the treatment (similar terms: patient treatment or appropriate treatment), for instance drug administration. At the end of the process chain we identified patient discharge, which is similar to a transfer to another department or location, e.g. arrival in the operation theatre. The final report is often sent after discharge and documented in the information system; so we included the final reports as our last point in time. Table 1 shows the results of the consensus process to design a generic timeline for all TAT definitions derived from our literature review.

**Table 1:** Preferred Terms

**Nr**.	Preferred term	Similar terms/Synonyms
1	*Admission*	▪ Hospitalization
		▪ Arrival at department

2	*Order*	▪ Request

3	*Biopsy/Examination*	▪ Collection of specimen
		▪ Sampling
		▪ (Operation) procedure
		▪ Dialysis Treatment started
		▪ Anaesthesia started
		▪ Endoscopist enters room
		▪ Start image production

4	*Receipt of specimen in laboratory*	▪ Patient leaves examination room
		▪ Dialysis treatment finished

5	*Procedure completion*	▪ Result available
		▪ Completion of examination
		▪ X-ray completion
		▪ Image ready
		▪ Processed angiogram
		▪ Notification of a scan (teleradiology)
		▪ Blood component ready
		▪ Image production

6	*Interpretation*	▪ Image display in ward
		▪ First diagnosis available

7	*Dictation*	

8	*Transcription*	▪ Typing
		▪ Documentation system available

9	*Verification*	▪ Report signing
		▪ Review
		▪ Documentation finished

10	*Report Available*	▪ Final diagnosis in registry

11	*Delivery*	▪ Report Delivery
		▪ Faxing of Preliminary Report (teleradiology)
		▪ Arrival blood components
		▪ Arrival medication

12	*Physician views report*	▪ Physician views result

13	*Treatment*	▪ Appropriate treatment
		▪ Patient treatment
		▪ Medication administration

14	*Discharge*	▪ Transfer (internally/externally)
		▪ Issue of exit sheet

15	*Discharge Letter sent*	

The table shows preferred terms and their synonyms for 15 points in time. They were identified through a consensus process while analysing starting and end points of the reviewed TAT definitions. These terms were used to illustrate the definitions on a generic timeline.

### Turnaround times in different clinical domains

#### Domain "Laboratory"

In the laboratory domain we identified 19 different TATs starting from the arrival in an emergency department to the reporting of the results and ending with the ordering of a form to the final report. The most common definition is the laboratory TAT beginning with the receipt of a specimen in a laboratory until the availability of the result, which was measured in 25 articles of this review. In 9 articles times were measured from the ordering of the results to their posting. Most references started from the order or from the receipt in the laboratory and nearly all references contained the interval between the receipt and the availability of the results. The points in time "interpretation", "dictation" and "transcription" were not covered.

#### Domain "Radiology"

In the radiology domain 11 different time intervals were found in our literature review. The most common definition was report TAT, which was measured from the X-ray completion until the availability of the radiology report in the HIS (10 articles). Four of these definitions concerned dictation-, typing- and signing-process, which ranged from X-ray completion until delivery of results (6 papers). The next frequent TAT definition was from radiology request to X-ray completion (5 papers). Three papers defined a TAT from radiology request to the availability of the report. Time intervals concerning the viewing of results or the following treatment were found in none of the analysed papers.

#### Domain "Whole Clinic and others"

This domain contains emergency department, medication and processes covering the whole clinic. We identified 21 different TAT definitions, of which the most commonly used (5 articles) spanned the entire stay in a hospital from admission to discharge or transfer. All other definitions were only used in one or two articles and addressed processes in different clinical departments.

**Figure 2:**
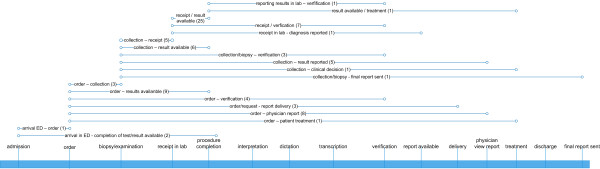
**Turnaround time definitions in the laboratory domain**. All identified definitions are illustrated with their starting and end points on a generic timeline. The number in brackets shows the number of papers in which the respective definition was used.

**Figure 3:**
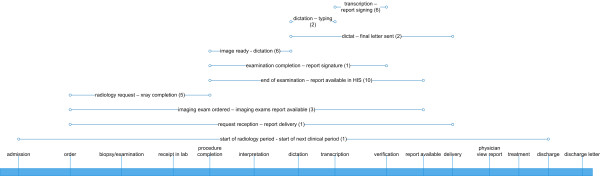
**Turnaround time definitions in the radiology domain**. All identified definitions are illustrated with their starting and end points on a generic timeline. The number in brackets shows the number of papers in which the respective definition was used.

**Figure 4:**
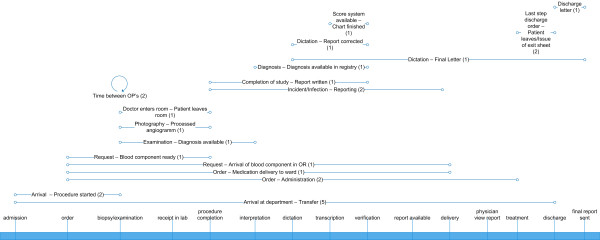
**Turnaround time definitions in the whole clinic and all other domains**. All identified definitions are illustrated with their starting and end points on a generic timeline. The number in brackets shows the number of papers in which the respective definition was used.

When comparing Figures 2, 3 and 4, it becomes evident that some definitions from the laboratory and radiology domain with the same starting and end points were used in a large number of publications (e.g. "time from the receipt to the availability of results" used in 25 studies). Such frequently used definitions, suited for comparative process analyses and benchmarking, could not be found in other domains

## Discussion

This review shows that TAT can be understood and measured in many different ways. In the laboratory domain, for example, the need to extend the early TAT definitions has existed since 1994, when Friedmann introduced the term "laboratory information float" to include the time when the result is available for the treating physician [146]. TAT is not a standardised measure and our results clearly show that there are many different time intervals, which all claim to be a TAT. There are also time intervals with the same starting and end points but all have different names. For benchmarking and optimisation of clinical processes the same understanding of TATs is crucial. Therefore, a consensus about these terms is necessary and should be agreed upon. Based on our review we propose the following definitions as described in table 2.

**Table 2:** Proposed turnaround time definitions

**Abbr**.	Long text	Start	End	Description
TAT	Turnaround time			Generic term for clinical processes

LTAT	Laboratory turnaround time	Receipt of specimen	Results available	Specific term for laboratorial processes

MTAT	Medication turnaround time	Request	Patient delivery	Includes drug turnaround time, and pharmacy turnaround time

ITAT	Imaging turnaround time	Request	Images available	Specific term for processes concerning images

RTAT	Report turnaround time	Request	Report available	Generic term for processes concerning all types of reports

TTAT	Total turnaround time	Request	Results available	Generic term for all kinds of hospital processes

This table proposes domain-specific and generic terms for TAT with defined starting and end points.

For the laboratory domain a similar approach to achieve a consensus was proposed by Ervasti et al., who emphasize the importance of the whole process and define TAT from a patient's point of view [147]. By introducing six TAT concepts based on four points in time (arrival, order, receipt, and report availability), the authors differentiate between the diagnostic TAT (arrival-to-report), the clinical TAT (arrival-to-order) and the laboratory TAT (order-to-report).

The measurement of TATs varies as much as their definitions. One study drew the data directly from their LIS [148] and thus used solely electronically available points in time. Process times can also be measured by shadowing medical staff and recording each working activity [149]. These different methods can result in different times and thus comparability between different studies is limited.

During our review of TATs we identified that times were measured in different units ranging from seconds to minutes to hours to days. Comparing different articles we found that the same definitions were measured in different units. As it did not seem relevant for our purpose we did not further evaluate these time units.

The idea of an abstract process is described by Kujala et al. who compare a patient episode with a customer order-to-delivery chain in industry [150]. Sinreich described a unified process chart from hospitalisation until discharge in 56 steps and defines 96 patient TATs to identify processes with major waiting times [151]. Other studies only focus on a few important points in time, which are then defined as milestones to describe relevant processes and time intervals [152]. In our approach to summarise TAT, we selected 15 points in time to represent the identified starting and end points on a level of details which covers all important aspects of the different clinical domains and provides a unified view on these clinical processes.

### Further observation

While collecting relevant literature, we noticed that the number of published papers in a specific domain was somehow connected with the period of publication. Through our search strategy we found only five papers between 1971 and 1992. During the late 90s the publication rate goes up to five papers per year on average. Until 2004 it further increases to over 10 papers per year. With an unexpected big peak in 2005 of 22 papers and a down of only four papers in 2006 the rate continuously increases to about 12 papers per year on average at the end of our observation period in 2010. Before the year 2000 most of the papers were published in the laboratory domain. Until the end of 2009 the laboratory domain is still the most prominent one (about 45% of all published papers). Radiology and whole clinic, both started to become significant around the year 2000 and produce a similar amount of papers per year on average. Their publication rate is a little lower than the rate of the laboratory domain. Regarding Medication and Emergency Department the total number of published papers is relatively low.

Despite the focus on specific clinical domains in most papers there are also studies regardless of the domain like Mechanic et al., who from 1989 - 1998 analysed the time spent in general at a physician's office [153]. It also became evident that process times are often defined for clinical routine only, but it is equally important to measure the effects in clinical research such as time for patient recruitment in clinical trials [154]. As clinical processes are becoming more and more comprehensive, publications in this area might increase in the coming years.

An important aspect that arises during the analysis of TAT is to clarify the purpose behind measuring or optimizing process times, for which there are different motivations. The most common background and motivation is to reduce the waiting time for patients, so that the treatment can be started earlier or the patient can be discharged earlier. Sometimes this can decrease costs for the hospital due to reduced redundant work and process streamlining [2].

Some authors focus their analysis of timelines on physician satisfaction [155], whereas others concentrate on the cost saving aspect [34]. A different approach is suggested by Holland et al., who argue that the TAT outlier percentage is a better method of benchmarking laboratory performance [156,157].

TAT are also commonly used to evaluate changes in certain processes (e.g. organizational changes, implementation of new information systems) by measuring the time needed before and after the intervention. However, it is difficult to directly compare these results, because of differences in the processes.

Other studies mentioned TAT changes as side effects. For example, Zardawi analysed the implementation of a quality assurance system that is primarily designed to reduce errors but they also found a reduction of TAT, even though it was not their main objective [158]. Also Ondategui-Parra et al. did not primarily analyse process indicators, but they put their emphasis on quality indicators like customer satisfaction [159].

Process times should not be the primary focus while analysing the quality of patient treatment. Yu and Gupta for example point out that the speed of analysing and reporting is not directly related to the quality of the interpretation [160]. Bewtra states that instead of further reducing TAT, a reasonable limit should be agreed on, because often patients do not benefit from the reduced TAT and it may even have negative effects on quality, teaching needs and the welfare of laboratory personnel [161].

### Limitations and strengths

While extracting the different TAT definitions we did not consider the context and the underlying research questions of the respective articles in detail. Although we know that this is relevant, a discussion with regard to content need to be based on consistent definitions. As there are a lot of different points in time that may all be relevant in certain studies, it is evident that there are also different TAT definitions. Nevertheless, a classification through standardised wording, the use of existing definitions and the awareness that TAT may not be a well-defined measure can contribute towards better comparability.

The main purpose of TAT is to describe workflows and perform quantitative analyses of processes. In this review we identified 162 TAT definitions which were used for different purposes. Some TATs are not precisely defined and many researchers do not use existing definitions while measuring their own workflows. This makes a comparison of processes very difficult. The illustration of different definitions on a generic timeline may help to categorize own research questions. The proposed generic terms makes it easier to differentiate between processes and sub-processes.

## Conclusion

Although measuring TAT is a common method when analysing clinical process times, it is difficult to compare studies, because there are many different definitions in use. In laboratory and radiology a considerable number of studies apply comparable TAT definitions. The mapping of TAT definitions to a generic timeline in order to facilitate benchmarking is feasible.

## Abbreviations

CPOE: Computerised physician order entry; HIS: Hospital information system; ITAT: Imaging turnaround time; LTAT: Laboratory turnaround time; MTAT: Medication turnaround time; RTAT: Report turnaround time; TAT: Turnaround time; TTAT: Total turnaround time;

## Competing interests

The authors declare that they have no competing interests.

## Authors' contributions

BB, FF and VT collected literature, summarized TAT definitions and wrote the manuscript. MD critically revised the manuscript. All authors read and approved the final manuscript.

## Pre-publication history

The pre-publication history for this paper can be accessed here:

http://www.biomedcentral.com/1472-6947/11/34/prepub

## Supplementary Material

Additional file 1 **TAT Definitions**. This file contains the full table with all TAT definitions and their references. Click here for file
